# Impact of two rounds of praziquantel mass drug administration on *Schistosoma mansoni* infection prevalence and intensity: a comparison between community wide treatment and school based treatment in western Kenya

**DOI:** 10.1016/j.ijpara.2016.01.006

**Published:** 2016-06

**Authors:** Isaac O. Onkanga, Pauline N.M. Mwinzi, Geoffrey Muchiri, Kennedy Andiego, Martin Omedo, Diana M.S. Karanja, Ryan E. Wiegand, W. Evan Secor, Susan P. Montgomery

**Affiliations:** a Center for Global Health Research, Kenya Medical Research Institute, P.O. Box 1578-40100, Kisumu, Kenya; b Division of Parasitic Diseases and Malaria, Centers for Disease Control and Prevention, 1600 Clifton Rd, N.E., Atlanta, GA 30329, USA

**Keywords:** Schistosomiasis, *Schistosoma mansoni*, Mass drug administration, Praziquantel, School based treatment, Community wide treatment, Prevalence

## Abstract

This study compared the effectiveness of the community-wide treatment and school-based treatment approaches in the control of *Schistosoma mansoni* infections in villages with ⩾25% prevalence in western Kenya. Stool samples from first year students, 9–12 year olds and adults (20–55 years) were analyzed by the Kato–Katz technique for *S. mansoni* eggs. After two rounds of treatment, *S. mansoni* prevalence and intensity levels significantly declined in both treatment approaches. Prevalence comparisons between the two approaches did not show any significant differences following treatment. However, infection intensity levels in the 9–12 year old school-attending pupils were significantly higher in the community-wide treatment arm than in the school-based treatment arm. Nevertheless, significant reductions in *S. mansoni* infection prevalence and intensity levels were achieved among school-age children regardless of the treatment approach used.

## Highlights

• Comparison of mass drug administration approaches for schistosomiasis was performed.
• The study presents results of a mid-term analysis of a 5 year study.
• School-based and community-wide distribution yielded a similar prevalence decrease.
• School-based distribution had a greater impact on intensity of infection.

## Graphical abstract

**Figure:**
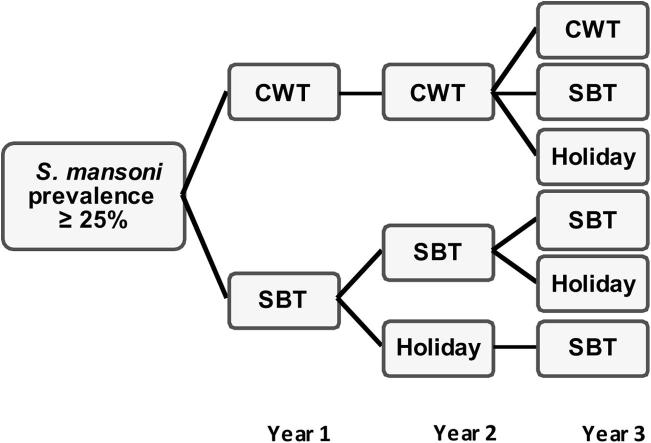

## 1 Introduction

Schistosomiasis remains one of the most important water-based diseases in the world, with an estimated 247 million people infected in 74 countries, 85% of whom are living in sub-Saharan Africa (World Health Organization, 1993, World Health Organization, 2002, Chan et al., 1994, Chitsulo et al., 2000). In Kenya, over 6.14 million people are estimated to have schistosomiasis with the highest infection rates found in adolescents (Grzych et al., 1987, Karanja et al., 1997, Karanja et al., 1998). Overall, the prevalence of schistosomiasis in endemic areas of Kenya ranges from 5% to 100%, contributes to significant morbidity (Ouma et al., 2001, Mwinzi et al., 2004, Odiere et al., 2012, Samuels et al., 2012) and is very high along the shores of Lake Victoria (Handzel et al., 2003, Standley et al., 2010). Morbidity associated with schistosomiasis includes under-nutrition, anaemia, chronic pain, diarrhea, exercise intolerance, growth stunting and cognitive impairment (Booth and Bundy, 1992, Stephenson et al., 2000). Much of the morbidity is reversible and can be controlled with treatment using praziquantel (PZQ) (Taylor-Robinson et al., 2007, Keiser and Utzinger, 2008).

Prevalence and intensity of infection are the key indicators currently used to measure the burden of schistosome infection in a given community. The number of eggs per gram (EPG) of feces provides a relative measure of infection intensity and is a key indicator of transmission dynamics within communities as well as the risk of morbidity among individuals (Anderson and May, 1985). Assessment of infection intensity requires quantitative laboratory methods that are time consuming. Prevalence of infection is the more easily collected indicator and is used by the World Health Organization (WHO) to provide guidelines for scope and frequency of mass treatment with PZQ (World Health Organization, 2013). PZQ is an effective, safe, single-dose treatment for schistosomiasis with few adverse events in uninfected individuals, making it appropriate for mass drug administration (MDA) for schistosomiasis control (Danso-Appiah et al., 2008).

In 2001, the 54th World Health Assembly endorsed resolution 54.19 to promote preventive measures, ensure treatment and mobilize resources for the control of schistosomiasis and soil-transmitted helminths (STHs) (Kabatereine et al., 2006). This resolution helped to increase interest from global sponsors and governments in endemic regions to control schistosomiasis and other neglected tropical diseases (NTDs) and to establish national action plans. Examples include Uganda and Tanzania in 2003 (Guyatt et al., 2001, Kabatereine et al., 2006, Kabatereine et al., 2007). In these countries, the main strategy for the delivery of anthelmintics was through the school system, an approach that reduces both infection and morbidity in a cost-effective manner as well as enhances educational outcomes in the treatment area (Guyatt et al., 2001, Miguel and Kremer, 2004, Kabatereine et al., 2007). However, treating only school children leaves out other members in the community who are also at risk of morbidity from infection and who could contribute to continued transmission. This includes out-of-school children, older children and adults as well as occupationally-exposed groups like car washers, sand harvesters, fishermen and fish handlers (Webbe and El Hak, 1990, Karanja et al., 1997, Karanja et al., 1998).

To also provide treatment for these groups, a community-based approach, where all members of the community are targeted for treatment, could be used. A multi-country study on community directed intervention (CDI) for onchocerciasis control demonstrated that community-based drug distribution can be effective and feasible for integrated delivery of different health interventions in rural Africa (Massa et al., 2009a, Massa et al., 2009b). However, direct comparison studies of school and community based approaches or combination strategies in different settings have not been performed, particularly in Kenya. Data are needed to inform the most effective control strategies and provide evidence for the most cost efficient deworming frequencies, for example, how often MDA should be conducted.

It is in this context that we are working with the Schistosomiasis Consortium for Operational Research and Evaluation (SCORE) to evaluate different MDA approaches with PZQ in areas of western Kenya with high prevalence (⩾25%) of *Schistosoma mansoni* infections in school age children. In a 5 year study, PZQ is delivered either through school-based treatment (SBT) or community-wide treatment (CWT). The two approaches involve regimens of drug treatment with varying treatment frequency. We report changes in *S. mansoni* prevalence and egg intensity levels after two rounds of MDA with reported treatment coverage rates. From the experience of two rounds of MDA, we also gained insight on how to improve MDA delivery.

## 2 Materials and methods

### 2.1 Study site, population and design

This study was conducted in eight districts in Nyanza province stretching approximately 300 km along the Kenyan shores of Lake Victoria. A total of 150 villages lying within 5 km of the lake shore were enrolled. Over 96% of the population in the study area are members of the Luo community. Although fishing is the main commercial activity in the villages, the majority of the residents are subsistence farmers. All residents in the area are at risk of infection with schistosomiasis due to frequent water contact in Lake Victoria. No PZQ MDA activities had been conducted in the area prior to 2011 when the study was initiated as the Kenya national program had yet to implement PZQ distribution. However, one albendazole MDA for STHs had been conducted in 2009.

**Fig. 1:**
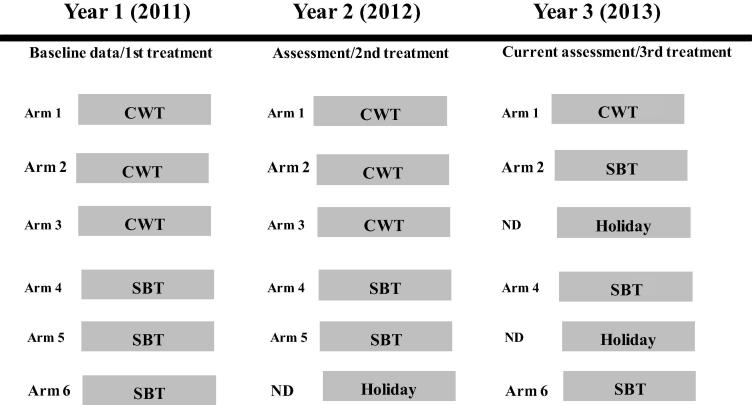
Schistosomiasis Consortium for Operational Research and Evaluation study design. Communities were randomised into six intervention arms. CWT, community-wide treatment; SBT, school-based treatment. ND, not done, indicating that stool samples were not collected for that arm because there was no treatment scheduled for that year.

Following an eligibility survey of 13–14 year olds to identify villages with ⩾25% prevalence, 150 communities were selected and randomised into one of six study arms, 25 villages per arm (Fig. 1). In arms 1–3 (CWT), community health workers (CHWs) were used to distribute drugs by going house-to-house each year during April, which is the school vacation period when students are most likely to be at home. CHWs provided PZQ for all persons who were eligible (>4 years of age and/or >94 cm tall). For persons who were present in the home, CHWs directly observed treatment. For persons who were not at home, the appropriate number of tablets were left. In arms 4–6 (SBT), health teachers were engaged to deliver PZQ to the children during February and March of each year and directly observed the students swallowing the treatment. Efforts were made to invite school age children who were not attending school to also participate in the MDA.

### 2.2 Study approval and involvement

The study was reviewed and approved by the National Scientific and Ethical Review Committees (ERC) of the Kenya Medical Research Institute (KEMRI) and the Institutional Review Board of the Centers for Disease Control and Prevention, USA, which deferred to the KEMRI ERC. Permission to conduct the study was obtained from the Ministry of Education, Ministry of Public Health and Sanitation and the Nyanza Provincial Administration, Kenya. Sensitisation of community members and school teachers was conducted to explain the purpose of the study and to gain support to test school children and adults in the community. CHWs and school health teachers were invited to training workshops where they were instructed in the collection of stool, use of the treatment dose pole, how to maintain drug administration records, and how to recognise serious adverse events (SAEs) associated with treatment. Consent documents and participation information were provided to potential participants in the language they understood best (English or Dholuo). Consent and assent for study participation and treatment were obtained from the parents or guardians and from the children, respectively.

### 2.3 Parasitological assessment

For the baseline assessment, 100 randomly selected 9–12 year old pupils and 100 first year students (children attending their first year of schooling) in each village were requested to provide stool samples. The 9–12 year olds provided three stool samples on three consecutive days while the first year students provided only one stool sample. In each community, 50 adults were randomly selected and, following enrollment and informed consent, asked to provide one stool sample. A greater number of samples were obtained from 9 to 12 year olds because the main goal of the study was to assess the impact of different MDA strategies on this age group. Sampling of first year students was performed to test if the MDA approaches had different impacts on the force of transmission and adults were sampled to monitor for any global changes that may be affecting the study village. However, as these were secondary questions and resources were limited, only one stool sample was collected from first year students and adults. Similarly, while stool samples were obtained from 9 to 12 year old students every year, according to the harmonised SCORE protocol, first year student and adult samples were only scheduled for the first (baseline), third (after two treatments), and fifth (after four treatments) years of the study. Each participant was issued empty stool cups and other sanitary necessities such as tissue paper and a scoop-stick, and the procedure for safe stool collection was explained. Stool sample collection took place over the course of 1 week in any one school or community by a team of one to two technicians with the help of a health teacher or CHW.

Stool samples were then transported to laboratories at KEMRI Center for Global Health Research (KEMRI-CGHR) and the Ministry of Health’s Division of Vector-Borne Diseases (DVBD) laboratory, Kisumu, where they were processed. Each stool sample was analyzed in duplicate by the Kato–Katz technique for eggs of *S. mansoni*, *Ascaris lumbricoides* and *Trichuris trichiura* by experienced examiners. The intensity of infection was calculated and egg data were recorded in booklets. After baseline stool sample collection in year 1, MDA with PZQ was conducted. In year 2, stool samples (three per participant) were only collected from 9 to 12 year olds in the village schools, and only in Arms 1–5 as Arm 6 was not receiving treatment that year. In year 3, stool samples were collected from first year students, 9–12 year olds, and adults in arms 1 and 2 (CWT) and from first year students and 9–12 year olds in Arms 4 and 6 (SBT). In years 2 and 3, new random samples of pupils and adults were recruited and requested to provide consent/assent to be evaluated for *S. mansoni* and STH infection. Stool collection, Kato–Katz slide preparation and microscopic examination to determine egg counts were performed as in the baseline survey.

### 2.4 MDA with PZQ and treatment coverage assessment

Treatment with PZQ as a single dose of 40 mg/kg was delivered using the WHO dose pole (Montresor et al., 2001). Usual WHO PZQ treatment exclusion criteria were applied: children under 4 years of age or under 94 cm in height were not treated. In villages receiving CWT, drugs were distributed door-to-door by CHWs during school holidays in an attempt to treat all members of the community. Consent to include a home compound in the treatment was sought from the compound head, but individuals were free to decline treatment. From the population census conducted by the CHWs, coverage in CWT villages was estimated by comparing the number of people (both adults and school age children) who received PZQ as recorded by CHWs with the eligible population in that particular village.

To evaluate coverage reported by CHWs, the study team conducted a household survey in all 75 villages in the CWT arms in the second year of the study. A list of households in a particular village was obtained from the population census earlier conducted by the CHWs in all CWT villages. Average village size was shown to be approximately 198 households, with the smallest village consisting of 40 households and the largest village consisting of 1200 households. In estimating treatment coverage in a particular village, the households were first ranked randomly, and depending on the size of the village, 15 or 30 households were selected for the household survey. Treatment coverage was then determined as the proportion of people (both adults and school age children) who reported they received PZQ compared with the total eligible population of the selected households in a particular village.

For villages assigned to SBT, primary school pupils were administered PZQ by trained school health teachers. The names of children who received PZQ were recorded in the treatment booklets and their number compared against the school enrollment list/eligible population to estimate treatment coverage.

According to SCORE goals, we targeted treatment coverage of ⩾90% in SBT arms and at least 75% in CWT arms. CHWs and school health teachers documented treatment coverage and reasons for the success or failure to meet the targeted coverage rate in treatment booklets. The information was then collected during CHW and health teacher feedback sessions.

### 2.5 Data collection and analysis

Field data were collected using barcoded master lists in which age, sex, class, school, village, parental consent and child assent, and number of stool samples and respective collection dates were recorded. In the laboratory, egg data were recorded in barcoded results booklets. Consistent with WHO guidelines, individuals with EPG <100 were considered to have low intensity infections, those with 100–400 EPG were considered to have moderate intensity infections, and persons with >400 EPG were considered to have high intensity infections. MDA data were recorded in treatment and drug tracking books. All the data from master lists and results booklets were then entered into smart phones installed with EpiCollect software for submission to the central server hosted at the Imperial College, London, UK. Data were also entered in Microsoft Excel spreadsheets and maintained on local desktop computers for back-up and analysis purposes.

Prevalence was assessed using a generalised linear model (GLM) with generalised estimating equations (GEE) (Liang and Zeger, 1986) to account for correlation between participants from the same community or school. Study arm, year and an interaction between the two were included as independent variables. A Poisson distribution was assumed for the outcome, allowing for results to be reported as prevalence ratios (PR).

Similar to the prevalence models, a GLM with GEE was used to model infection intensity. A gamma distribution was found to best match the EPG distribution, especially since infected and non-infected participants were included in the model. One was added to each participant’s EPG value since the gamma distribution is only defined for positive real numbers. This was then subtracted from the final parameter estimates. Comparisons are reported as arithmetic mean ratios (AMR), a ratio of the two estimated mean EPGs in the comparison.

All statistical analyses were performed in SAS version 9.3 (SAS Institute, Inc., Cary, NC, USA) using PROC GENMOD and the 5% level of significance. Where appropriate, post-hoc contrasts were used to estimate prevalence or intensity and make year to year comparisons in single or across multiple arms.

## 3 Results

### 3.1 MDA treatment coverage in first and second rounds of treatment

Table 1 summarises treatment coverage as reported by the CHWs and health teachers and from the household survey in year 2. In the CWT arm, 75 villages participated in both rounds of treatment. In the first round, CHWs reported having given treatment to 24,118 people out of an eligible population of 28,694, representing a coverage rate of 84.1% (95% confidence interval (CI) = 83.2–89.4%). In the second round, they reported treatment of 25,399 people out of an eligible population of 29,011 representing a coverage rate of 87.6% (95% CI = 85.3–91.1%). In both rounds of treatment, 65 (86.7%) villages achieved the 75% treatment coverage mark. The independent household survey, conducted after the second round of treatment, revealed that treatment was given to a total of 3781 people out of an eligible population of 6055, representing a coverage rate of 62.4% (95% CI = 58.7–67.2%) with only 25 (33.3%) villages attaining ⩾75% coverage.

In the SBT arms, 75 and 51 villages were scheduled for the first and second rounds of treatment, respectively. In the first year, treatment was given to 22,406 school-age children out of an eligible population of 26,487, representing a treatment coverage of 84.6% (95% CI = 82.9–87.0%). In the second round, treatment was given to 15,984 school age children out of an eligible population of 17,796 representing a coverage rate of 89.8% (95% CI = 86.2–91.4). In the first and second rounds of treatment, 28 (37.3%) and 22 (43.1%) schools achieved the 90% treatment coverage goal.

### 3.2 *Schistosoma mansoni* infection prevalence and intensity in years 1–3 in CWT and SBT

Prevalence and intensity data for *S. mansoni* infections are presented in Table 2 and relative changes over time are shown in Table 3. Comparison of baseline data indicated that there were no significant differences between the arms or treatment strategies, indicating that the randomisation was effective (data not shown). Not surprisingly, the highest infection levels occurred in 9–12 year olds, followed by the adults and the first year students. Among 9–12 year olds, significantly lower prevalence levels were recorded in year 2 than in year 1 both in the CWT (PR = 0.85, 95% CI = 0.80–0.90, *P* < 0.001) and SBT (PR = 0.71, 95% CI = 0.66–0.78, *P* < 0.001) arms. Similarly, year 3 prevalence levels in all the study groups were significantly lower than those in year 1 for both treatment approaches (9–12 year olds (CWT (PR = 0.77, 95% CI = 0.67–0.88, *P* < 0.001, and SBT PR = 0.56, 95% CI = 0.47–0.67, *P* < 0.001), first year students (CWT PR = 0.61, 95% CI = 0.47–0.79, *P* < 0.001 and SBT PR = 0.65, 95% CI = 0.48–0.87, *P* = 0.004) and adults (CWT PR = 0.37, 95% CI = 0.28–0.47, *P* < 0.001)). Comparisons between year 3 and year 2 prevalence levels were only done in the 9–12 year olds and only the CWT arm recorded significantly lower prevalence levels in year 3 than year 2, PR = 0.84, 95% CI = 0.76–0.93, *P* < 0.001. For the intensity comparisons, in the 9–12 year olds, only the SBT arm recorded significantly lower intensity levels in year 2 than those in year 1, AMR = 0.46, 95% CI = 0.41–0.52, *P* < 0.001. However, both treatment approaches recorded significantly lower infection intensity levels in year 3 than those in year 1 (CWT AMR = 0.77, 95% CI = 0.62–0.94, *P* = 0.01, and SBT AMR = 0.37, 95% CI = 0.27–0.51, *P* < 0.001). However, first year students and the adult categories’ infection intensity levels in year 3 were only significantly lower in the CWT arm when compared with those in year 1 (first year students AMR 8 0.60, 95% CI = 0.41–0.89, *P* = 0.01 and adults AMR = 0.42, 95% CI = 0.26–0.67, *P* < 0.001). Comparisons between years 3 and 2 intensity levels included only the 9–12 year olds and only the CWT arm recorded significantly lower infection intensity levels in year 3 than in year 2 (AMR = 0.77, 95% CI = 0.62–0.95, *P* = 0.01).

### 3.3 Prevalence and intensity comparisons between CWT and SBT arms in each study year

Although schistosomiasis prevalence levels were generally lower in the SBT arms after treatment in all age groups, comparisons of the prevalence levels among the 9–12 year olds between the CWT arms and the SBT arms did not show any significant differences across the three years of the study (Table 4). Similarly, in the first year students and adults, no significant differences were observed between the two treatment approaches at baseline for either age group or in year 3 for the first year students. The intensity levels in the 9–12 year olds were not significantly different between the two treatment approaches at baseline but in both years 2 and 3, the intensity levels were significantly higher in the CWT arms than in the SBT arms (year 2 AMR = 1.91, 95% CI = 1.25–2.93, *P* = 0.003, and year 3 AMR = 2.37, 95% CI = 1.20–4.69, *P* = 0.01). For the first year students and adults, the infection intensity levels were not significantly different between the two treatment approaches at baseline or for the first year students in year 3.

## 4 Discussion

Depending on the prevalence of schistosomiasis infection in a given community, WHO recommends MDA delivery using either a school-based or community-wide approach. In this study, the effectiveness of SBT and CWT was compared for the control of *S. mansoni* infections in villages with an initial prevalence ⩾25%. Parasitological assessment following two rounds of treatment revealed that both CWT and SBT treatment approaches had a significant impact on the prevalence and intensity of schistosomiasis. Comparisons of the effect on prevalence between the two approaches did not show any significant differences. Thus, SBT and CWT were equally effective in reducing the prevalence of *S. mansoni*. By contrast, the SBT arms demonstrated significantly higher reductions in infection intensity than the CWT arms after 1 and 2 treatments among 9–12 year olds.

A number of villages failed to attain the targets set by the SCORE protocol, even after follow-up treatment efforts in areas that had earlier recorded low treatment coverage. Although the CHWs’ reports indicated the CWT arm had higher coverage than the SBT arm, assessing coverage was problematic in the CWT villages as artificially high coverage rates were reported by the CHWs. This was partly due to the fact that the CHWs had conducted a census ahead of the treatment exercise and used those data to estimate coverage from their records; however, in some cases the census was not necessarily based on all compounds in villages, depending on the diligence of the CHW.

Some of the factors that might have influenced the increased impact on infection intensity in the SBT arm include that children in schools are already congregated, higher compliance during screening and treatment may be linked to direct supervision by school health teachers, (Guyatt and Tanner, 1996) and efforts to conduct follow-up treatments to reach those who were absent during the initial treatment exercise are more efficient. However, some schools recorded coverage rates that were less than the SCORE protocol target. Factors that contribute to lower coverage in such schools should be evaluated to assist in development of approaches to improve coverage for better control of schistosomiasis (Touré et al., 2008).

When we visited the schools in the CWT arms for parasitological assessment in the second year of the study, some teachers questioned why certain students had not received treatment in the community drug distribution. To address this concern, a household survey was conducted after the second round of treatment. It revealed that actual coverage rates and the number of villages with ⩾75% treatment coverage in the CWT arms were significantly lower than what had been reported by the CHWs and that treatment coverage in the CWT arms may have been substantially lower than that in the SBT arms. Thus, the higher apparent impact of SBT on infection intensity compared with CWT may simply be a function of the treatment coverage challenges in the CWT arm.

Poor understanding of village boundaries, difficulties in reaching community members, lack of supervision during treatment and the fact that this was the first MDA in this area are some of the reasons that might explain the lower performance in the CWT arm. A similar scenario was reported in the Philippines where village leaders were primarily responsible for community mobilisation and the extent of their support for the program was unclear and possibly incomplete, as less than 50% coverage was achieved (Tallo et al., 2008). Many studies have shown significant differences between reported coverage and household survey coverage rates (Mwinzi et al., 2012). Use of the reported treatment coverage data with the possibility of exaggeration by the CHWs and health teachers constitutes a major limitation in our study as acceptance of these data at face value may have introduced bias in our findings. After we identified the discrepancies between the reported coverage and household survey data in the CWT arms, CHW training prior to the next round of MDA focused on improved data collection and accurate reporting of coverage rates. This included public education and awareness efforts aimed at community mobilisation conducted before commencement of MDAs, such as radio announcements, road shows, notices and barazas (community gatherings convened by the village chief) (Omedo et al., 2014). Treatment coverage rates attained in our study differ from coverage reported with studies conducted in Uganda (Ndyomugyenyi and Kabatereine, 2003), and Tanzania (Massa et al., 2009a), where higher treatment coverage rates were achieved in the community approach compared with the school approach, indicating that treatment compliance levels may differ among countries. Further qualitative research in our study is ongoing to identify treatment compliance issues at the community and school levels and ways to improve it.

Reported impact of the two approaches, SBT compared with CWT, on prevalence and intensity of schistosomiasis has not been consistent across studies or countries. Our findings on the reduction in schistosomiasis prevalence and intensity levels differed from studies conducted in Uganda (Zhang et al., 2007) and Burkina Faso (Touré et al., 2008). In these two countries, the effect of PZQ treatment using the CWT approach was greater than using the SBT approach. However, our findings match those of another study from Tanzania on the effectiveness of CWT as an alternative approach for MDA delivery (Massa et al., 2009b).

In conclusion, our midterm evaluation findings from this 5 year study indicate that significant reductions in schistosomiasis prevalence and intensity levels were achieved regardless of the treatment approach used. However, the failure of some communities to attain treatment coverage targets set by the SCORE protocol and the threshold recommended by the WHO, together with the finding that *S. mansoni* prevalence and intensity levels still remained relatively high after two rounds of MDA, signify the need for intensified community mobilisation for increased participation, which will be critical to achieve better control of schistosomiasis. In high prevalence communities, it may also be necessary to provide MDA more than once a year.

## Figures and Tables

**Table 1:** Mass drug administration coverage in first and second rounds of treatment with praziquantel.

Treatment round	Arm	No. of villages surveyed (*n*)	Eligible population	Number treated	Coverage (%)	95% CI	Villages with ⩾75% CWT or ⩾90% SBT coverage, *n* (%)
First round	CWT	75	28,694	24,118	84.1^a^	83.2–89.4	65 (86.7)^a^
	SBT	75	26,487	22,406	84.6^a^	82.9–87.0	28 (37.3)^a^
Second round	CWT	75	29,011	25,399	87.6^a^	85.3–91.1	65 (86.7)^a^
	SBT	51	17,796	15,984	89.8^a^	86.2–91.4	22 (43.1)^a^
Household Survey		75	6055	3781	62.4^b^	58.7–67.2	25 (33.3)^b^

CWT, community wide treatment; SBT, school based treatment; CI, confidence interval.

a Coverage as reported by community health workers (CWT arms) or teachers (SBT arms);

b Coverage determined by the household survey.

**Table 2:** *Schistosoma mansoni* infection prevalence and intensity levels in years 1–3 in community wide treatment and school based treatment (SBT) arms.

Study year	Study group	Study arms		Prevalence (95% CI)		Mean EPG (95% CI)	
CWT	SBT	CWT	SBT	CWT	SBT
2011	First year students	1, 2, 3	4, 5, 6	26 (21–33)	29 (24–35)	53.0 (36.1–77.6)	54.1 (37.4–78.1)
	9–12 years	1, 2, 3	4, 5, 6	60 (55–66)	63 (58–68)	88.5 (70.0–111.9)	91.6 (72.1–116.4)
	Adults	1, 2, 3	4, 5, 6	42 (38–47)	46 (41–50)	61.7 (50.7–75.1)	73.0 (59.4–89.5)
2012	9–12 years	1, 2, 3	4, 5	51 (46–57)	45 (38–52)	79.3 (59.1–106.1)	41.0 (29.7–56.6)
2013	First year students	1	4	21 (14–32)	18 (12–28)	45.6 (25.3–81.5)	32.3 (16.3–79.6)
	9–12 years	1	4	48 (38–61)	34 (25–45)	76.9 (49.4–119.3)	31.8 (18.4–54.4)
	Adults	1	–	16 (12–22)	–	22.5 (12.2–40.8)	–

CI, confidence interval; EPG, eggs per gram.

**Table 3:** Prevalence and intensity comparisons between study years in community wide treatment and school based treatment arms.

Study group	Years compared	Study arm	Individual arms compared	PR (95% CI)	*P* value	AMR (95% CI)	*P* value
9–12 years	2012 vs. 2011	CWT	1, 2, 3 vs. 1, 2, 3	0.85 (0.80–0.90)	<0.001	0.90 (0.78–1.03)	0.12
		SBT	4, 5 vs. 4, 5, 6	0.71 (0.66–0.78)	<0.001	0.46 (0.41–0.52)	< 0.001
	2013 vs. 2011	CWT	1 vs. 1, 2, 3	0.77 (0.67–0.88)	<0.001	0.77 (0.62–0.94)	0.01
		SBT	4 vs. 4, 5, 6	0.56 (0.47–0.67)	<0.001	0.37 (0.27–0.51)	< 0.001
	2013 vs. 2012	CWT	1 vs. 1, 2, 3	0.84 (0.76–0.93)	<0.001	0.77 (0.62–0.95)	0.01
		SBT	4 vs. 4, 5	0.88 (0.75–1.03)	0.12	0.83 (0.57–1.22)	0.35
First year students	2013 vs. 2011	CWT	1 vs. 1, 2, 3	0.61 (0.47–0.79)	<0.001	0.60 (0.41–0.89)	0.01
		SBT	4 vs. 4, 5, 6	0.65 (0.48–0.87)	0.004	0.79 (0.34–1.82)	0.58
Adults	2013 vs. 2011	CWT	1 vs. 1, 2, 3	0.37 (0.28–0.47)	<0.001	0.42 (0.26–0.67)	<0.001

PR, prevalence ratio; AMR, arithmetic mean ratio; CI, confidence interval.

**Table 4:** Prevalence and intensity comparisons between community wide treatment and School based treatment arms in each study year.

Study year	Study group	Arms compared		PR (95% CI)	*P* value	AMR (95% CI)	*P* value
CWT	SBT
2011	First year students	1, 2, 3	4, 5, 6	0.90 (0.68–1.20)	0.48	0.98 (0.58–1.65)	0.94
	9–12 years	1, 2, 3	4, 5, 6	0.96 (0.85–1.08)	0.51	0.97 (0.69–1.35)	0.84
	Adults	1, 2, 3	4, 5, 6	0.93 (0.81–1.06)	0.27	0.85 (0.64–1.12)	0.25
2012	9–12 years	1, 2, 3	4, 5	1.15 (0.95–1.38)	0.15	1.91 (1.25–2.93)	0.003
2013	First year students	1	4	1.14 (0.62–2.10)	0.67	1.25 (0.48–3.26)	0.65
	9–12 years	1	4	1.42 (0.99–2.05)	0.06	2.37 (1.20–4.69)	0.01

PR, prevalence ratio; AMR, arithmetic mean ratio; CI, confidence interval.
